# The Dissolution Behavior of Feldspar Minerals in Various Low-Molecular-Weight Organic Acids

**DOI:** 10.3390/ma16206704

**Published:** 2023-10-16

**Authors:** Shao-Min Lin, Ya-Ling Yu, Ming-Feng Zhong, Huan Yang, Chen-Yang Zhang, Zhi-Jie Zhang, Yun-Ying Wu

**Affiliations:** 1 School of Materials Science and Engineering, Hanshan Normal University, Chaozhou 521041, China; 2 School of Materials Science and Engineering, South China University of Technology, Guangzhou 510641, China; 3 Chaozhou Branch of Chemistry and Chemical Engineering Guangdong Laboratory, Chaozhou 521041, China; 4 Guangdong Chaoshan Institute of Higher Education and Technology, Chaozhou 521041, China

**Keywords:** dissolution, feldspar, low-molecular-weight organic acids, crystal plane, nanostructures

## Abstract

Feldspar is a high-abundance mineral in the earth’s crust, and its natural weathering and dissolution processes are an important phenomenon on the earth’s surface. This study focused on the dissolution behavior of silicon (Si) and aluminum (Al) in feldspar minerals (microcline and albite) when exposed to low-molecular-weight organic acids (LMWOAs). Various analytical techniques, including atomic absorption spectrophotometer, X-ray diffraction, scanning electron microscope, and Fourier-transform infrared spectroscopy, were employed to investigate these processes. The results revealed that the concentration of Si and Al released from alkali feldspar increased after treatment with LMWOAs, exhibiting non-stoichiometric dissolution. The Si/Al release ratio from feldspar deviated from the expected value of three. Among the LMWOAs tested, oxalic acid was found to be more effective in dissolving aluminum, while citric acid showed greater efficacy in dissolving silicon. Notably, the composite acid demonstrated the highest capacity for feldspar dissolution, with values of 538 μM (Si) and 287 µM (Al) after treatment for 720 h, respectively. The dissolution data for Si and Al in the organic acid solution was fittingly described by a first-order equation, with high correlation coefficients (R^2^ ≥ 0.992). The characterization of feldspar powders indicated that the (040) crystal plane of feldspar was particularly susceptible to attack by organic acids. In the presence of these acids, the chemical bonds Si (Al)-O, Si-Si(Al), and O-Si(Al)-O shifted to higher wavenumbers. Additionally, the surface corrosion morphology of feldspar exhibited distinct nanostructures, which became more pronounced with increasing exposure time. It was also observed that the reactivity of feldspar increased over time. These findings provide valuable insights into the natural dissolution process of feldspar and offer a new perspective for the study of this phenomenon.

## 1. Introduction

Low-molecular-weight organic acids (LMWOAs) are ubiquitous in soils and sediments [1]. Due to the ability to strongly bind to mineral surfaces and complex metal cations in a solution of LMWOAs, LMWOAs such as lactate and oxalate are suspected to enhance mineral dissolution rates. They can react with minerals in the earth’s crust and are known to play an important role in the alteration and weathering reactions at the mineral–water interface [2].

The effect of LMWOAs on mineral dissolution has been widely investigated, since the important role of LMWOAs in the dissolution of minerals in the soil was reported [3,4,5,6,7]. Generally, different kinds of organic acids were selected to simulate the mineral (such as kaolinite, albite, microcline, quartz, and so on) dissolution and study the effect of time, temperature, pH, and organic acid species on the dissolution rate of Si and Al from the mineral [8,9,10,11] The research was mainly conducted by authors from the fired geochimica or cosmochimica, and low-concentrated LMWOAs were selected for simulating the chemistry weathering of feldspar. Blake and Walter [12] reported that in the solution of 0.5, 3, and 10 mM oxalate and citrate, the carboxylic acid species significantly enhanced the dissolution of Si and Al from orthoclase, while the dissolution of quartz did not increase with increasing organic acid concentration. Stillings et al. measured feldspar dissolution rates over the oxalic acid concentration of 0–8 mM and the feldspar compositional range of An0–An76, and suggested that oxalate played a role in the dissolution mechanism [13]. Cama and Ganor studied the dissolution rate of Al and Si release from kaolinite over the oxalate concentration range of 0.05~1.5 mM, and revealed that the dissolution of kaolinite is catalyzed by the simultaneous adsorption of two ligands on two neighboring edge alumino sites [14].

The release of Al and Si from minerals in the laboratory was found to be minimal in the presence of LMWOAs [9,15,16]. In order to replicate the natural geochemical environment, researchers utilized low concentrations of organic acids in the laboratory [17,18,19,20]. However, it is important to note that the laboratory environment cannot fully replicate the complexities of the natural world. As a result, the dissolution rate of minerals in the laboratory is significantly lower compared to that under geochemical conditions. In the natural geochemical environment, there are various living organisms such as plants or bacteria that can generate a wide range of low-molecular-weight organic acids [21,22,23,24,25]. Therefore, it is feasible for these organisms to release different LMWOAs onto the surface of minerals, creating a specific microzone environment with high concentrations of organic acids, and enhancing the dissolution rate of minerals.

Through extensive literature research, it was observed that little attention has been given to the dissolution of minerals in the presence of composite LMWOAs, and the changes in minerals under these conditions are rarely discussed. Hence, it is crucial to investigate the dissolution behavior of feldspars, which are abundantly distributed on the earth’s crust, under the influence of high concentrations of organic acids. This paper focuses on studying the dissolution kinetics of Si and Al from feldspar in different organic acid solutions. Additionally, it examines the alterations of the alkali-feldspar powders and discusses the dissolution mechanism.

## 2. Materials and Methods

### 2.1. Materials

The quartz and alkali feldspar were obtained from Guangdong Changlong Company Ltd. (Dapu, China) The mineral was crushed coarsely, and any apparent impurities were eliminated. They were then ground and wet-sieved through a 200-mesh sieve. To eliminate contaminants, the powders were cleaned three times with deionized water. The powders were then washed and dried at 105 °C.

### 2.2. Experimental Procedure

First, 4.00 g of the purified powder samples with a D50 of 10.06 μm, and 200 mL of the aqueous solution deionized water (blank sample), 40 mM oxalic acid (C_2_H_2_O_4_·2H_2_O, AR, Fuchen Chemical Reagents Factory, Tianjin, China), and 40 mM citric acid (C_6_H_8_O_7_·H_2_O, AR, Fuchen Chemical Reagents Factory, Tianjin, China) or composite acids (20 mM oxalic acid and 20 mM citric acid) was added to a 250 mL polypropylene bottle. The solution was adjusted to pH 4 using 0.05 M K_2_HPO_4_ (Xilong Science Co., Ltd., Shantou, China) and 0.05 M HCl (Xilong Science Co., Ltd., Shantou, China). The 2 mL chloroform had been added to each bottle. The mixtures were stirred at the speed of 500 r/min for 6 min. Then, each bottle was sealed and fully immersed in a thermostatic water bath at 25 °C. At regular intervals, the samples were stirred at the speed of 500 r/min for 1 min, then centrifuged and filtered through 0.45 μm nylon filters. The liquid phase was collected for later use. The solid phase in the centrifuge tube was washed several times with de-ionized water until the electrical conductivity of the dispersion decreased to 20 μScm^−1^. Finally, the filter was dried and stored for later use.

### 2.3. Characterization of the Sample

The feldspar mineral composition was checked by X-ray powder diffraction (X’pertPro, Panlytical corporation, EA Almelo, The Netherlands). The data were recorded at 40 kV and 40 mA with Cu kα (0.15418 nm) filtered radiation, and a curved graphite secondary monochromator covering 2θ between 10 and 90° with a step width of 0.02° 2θ and 0.1 s data collection per step. The measured powder was sieved through a 200-mesh sieve. The sample was then filled into a groove in the sample stage and compressed using a flat, smooth glass plate. Any excess powder outside the groove or above the sample plate surface was scraped off, and the sample was repressed until its surface became level and smooth, aligned with the sample plate surface.

Fourier-transform infrared spectroscopy (FT-IR) was used to investigate the feldspar. The FTIR spectra were recorded on a Vector 33 produced by Bruker Corporation (Billerica, MA, USA). In total, 64 scans were recorded between 4000 and 400 cm^−1^(resolution of 0.5 cm^−1^) using the KBr pellet method (2 mg of sample dispersed in 200 mg KBr).

The chemical composition of the raw sample was determined by X-ray fluorescence (XRF) analysis spectrometry using a PANalytical Axios PW4400 spectrometer (Almelo, The Netherlands), equipped with a rhodium X-ray tube running at 4 KW. The specific surface areas (SSA) were determined via BET measurements with nitrogen gas adsorption using FlowSorb III 2310 (Micromeritics, Norcross, GA, USA). Scanning electron microscopy (SEM) images of kaolinite were performed by a Quanta 200 apparatus (FEI, Hillsboro, FL, USA), using a secondary electron detector with an accelerating voltage of 5 kV, magnification of 14× to 300,000×, and working distance of 5 mm.

The concentration of Al in the supernatant was determined by atomic absorption spectroscopy (contrAA700, Analytik Jena AG, Jena, Germany). The concentration of Si was obtained by the Si molybdenum blue spectrophotometric method with a visible light spectrophotometer (722 N, Yoke Instrument Co., Ltd., Shanghai, China).

## 3. Results and Discussion

### 3.1. The Dissolution Kinetics of Al and Si from Alkali Feldspar in LMWOAs

**Figure 1:**
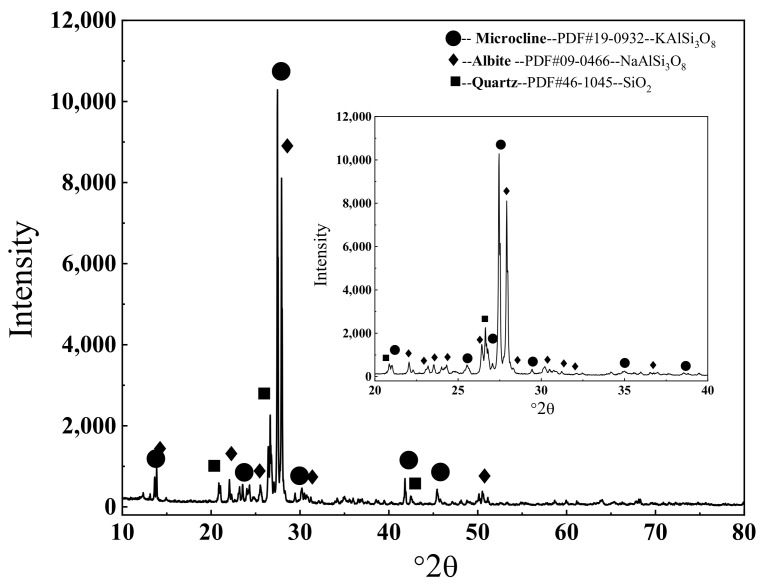
The XRD pattern of the minerals.

The chemical composition (Table 1) and the XRD pattern of the feldspar mineral (Figure 1) revealed that the main crystal phases in the alkali feldspar were microline (ICDD PDF 19-0932) and albite (ICDD PDF 09-0466), and trace amounts of quartz (ICDD PDF 46-1045) were identified.

**Figure 2:**
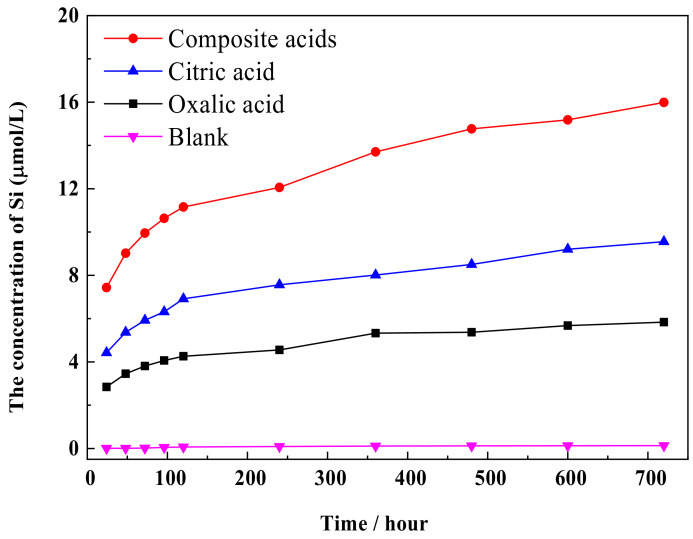
The concentration of Si released from quartz.

Figure 2 illustrates the concentration of Si released from quartz at pH = 4 over time. Without LMWOAs, the Si concentration remains below 16 μmol/L. In the presence of LMWOAs for 720 h, the Si concentration increases, reaching a value of 16 μm/mol. The order of LMWOAs in terms of Si extraction from quartz is as follows: composite acids > citric acid > oxalic acid > blank.

**Figure 3:**
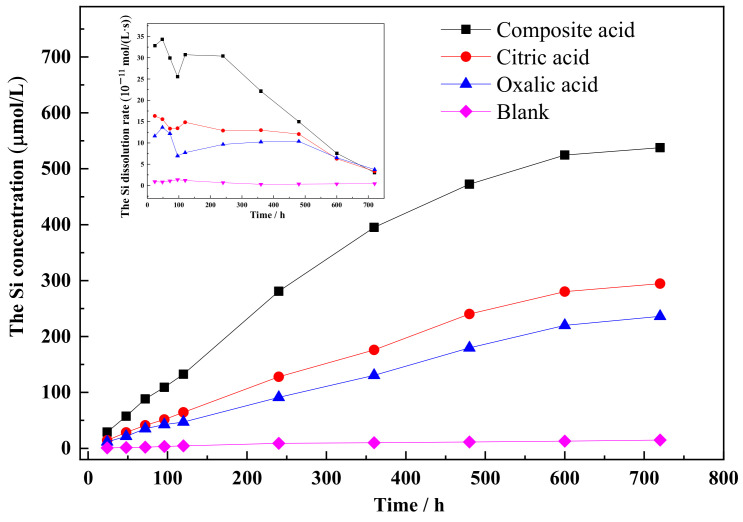
The concentration of Si released from the sample.

**Figure 4:**
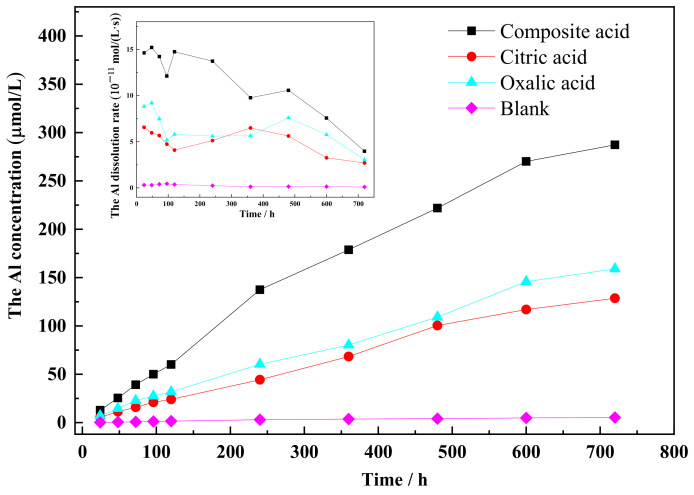
The concentration of Al released from the sample.

The dissolution curves of Si and Al show similar patterns, with an increase in dissolution concentration over time (refer to Figure 3 and Figure 4). The rate of increase in dissolution concentration is higher during the initial reaction time, followed by a decrease. LMWOAs also play a role in the extraction of Si from feldspar. The order of effectiveness is as follows: composite acid > citric acid > oxalic acid > blank. In contrast, the order of organic acid effectiveness in extracting Al is as follows: composite acid > oxalic acid > citric acid > blank. Comparing single organic acid solvents to composite organic acids, the dissolution concentration of Si and Al is lower in the former. The maximum values of Si and Al concentration are 538 μM and 287 μM, respectively. The concentration of Si released from the sample (comprising microcline, albite, and quartz) is much higher than that released from quartz (Figure 2). The data suggest that the Si present in the solution mainly originates from the feldspar in the sample.

**Figure 5:**
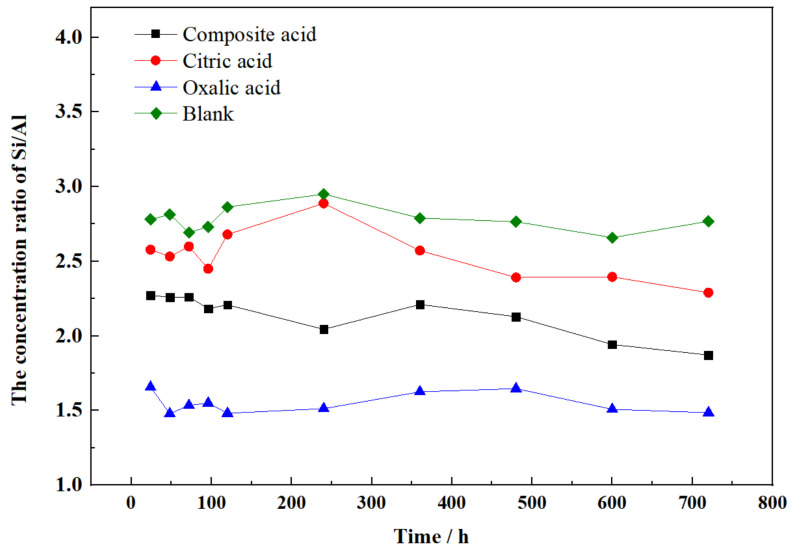
The concentration ratio of Si/Al released from the sample.

A lower Si/Al release ratio from the feldspar mineral (Figure 5) was noted, which deviated from the expected value of three in the chemical formula of the main crystal in feldspar mineral ((Na, K)AlSi_3_O_8_). This finding suggests that the release of an Al atom did not result in the simultaneous release of three Si atoms from feldspar. This disparity can be attributed to the higher bond energy of Si-O in the crystal lattice. In the presence of individual acids, the Si/Al ratio reached its minimum in oxalic acid and its maximum in citric acid. These results indicate that citric acid is more effective in extracting Si from feldspar compared to oxalic acid, while oxalic acid facilitates the extraction of Al from feldspar more readily than citric acid. The ratio of Si/Al released to solution indicates that the organic ligand plays a role in enhancing the dissolution of the feldspar [26].

To fit the dissolution concentration data of Si and Al release from feldspar (Table 2 and Table 3), three equations were employed: the parabolic diffusion equation (Equation (1)), the Elovich equation (Equation (2)), and the first-order equation (Equation (3)).

(1) $$C_{t}=a+bt^{1/2}$$

(2) $$C_{t}=a+bInt$$

(3) $$C_{t}=a(1-exp(-kt))$$

where $C_{t}$ is concentration of atoms (Si, Al) released from the mineral after dissolution for *t* (hour). a and b were the constant of the kinetic equation. k was the rate coefficient.

In the presence of low-molecular-weight organic acids (LMWOAs), the data fit well with a first-order equation (R^2^ ≥ 0.992). The order of the rate coefficients (k) for Si and Al dissolution was as follows: k (composite acid) > k (citric acid) > k (oxalic acid) > k (blank), and k (composite acid) > k (oxalic acid) > k (citric acid) > k (blank), respectively. These orders were consistent with the dissolution concentrations of Si and Al.

The results revealed that citric acid had a stronger ability to dissolve Si from feldspar compared to oxalic acid. Conversely, oxalic acid showed a stronger ability to dissolve Al from alkali feldspar compared to citric acid. The composite organic acid demonstrated the highest dissolving capacity for Si and Al when compared to the individual acids. This can be attributed to two factors: firstly, the composite organic acid consisting of oxalic acid and citric acid simultaneously attacked Si and Al sites in feldspar. Secondly, the substantial release of Si and Al from albite and microcline resulted in increased defects on the mineral surface, leading to the formation of new Si and Al sites.

### 3.2. The Changes of Alkali Feldspar after Treatment with LWMOAs

**Figure 6:**
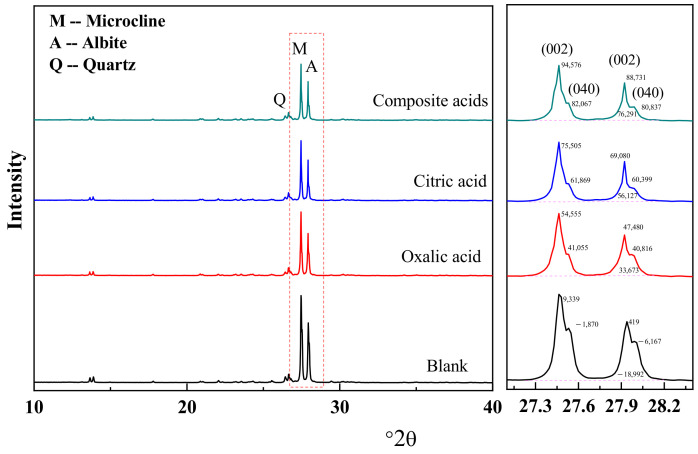
The XRD patterns of the sample in different LMWOAs.

The X-ray diffraction (XRD) results (Figure 6) indicate that microcline (PDF#19-0932) and albite (PDF#09-0466) were the predominant crystal phases in feldspar mineral, with only small quantities of quartz (PDF#46-1045) present. Upon treatment with LMWOAs, the XRD intensities of quartz remained relatively stable, while the intensities of the (002) and (040) reflections for albite and microcline decreased significantly. These findings suggest the effectiveness of LMWOAs in altering the crystal structure of albite and microcline.

Formula (4) states that the diffraction peak intensity of a crystal in a given sample is proportional to the square of the number of crystals per unit volume. Consequently, it can be inferred that the diffraction peak intensity of a specific crystal face has a positive correlation with the number of that crystal face present per unit volume. Therefore, a higher quantity of such crystal faces would result in a greater diffraction peak intensity. According to the XRD results, the organic acids have caused damage to the (002) and (040) crystal faces of albite and microcline.

(4) $$I={c\cdot P\cdot L\cdot N}_{c}^{2}\cdot {\left|F_{\left(hkl\right)}\right|}^{2}\cdot D\cdot M\cdot V\cdot$$

*c* is the constant unrelated to crystal structure; P is the polarization factor; L is the Lorenze factor; $N_{c}$ is the crystal cell number per unit volume, $F_{\left(hkl\right)}$ is structure factor; D is the Debye factor; M is the multiplicity factor; V is the effective volume of the sample involved in diffraction, which is related to the sample’s absorption coefficient (μ) and shape.

**Figure 7:**
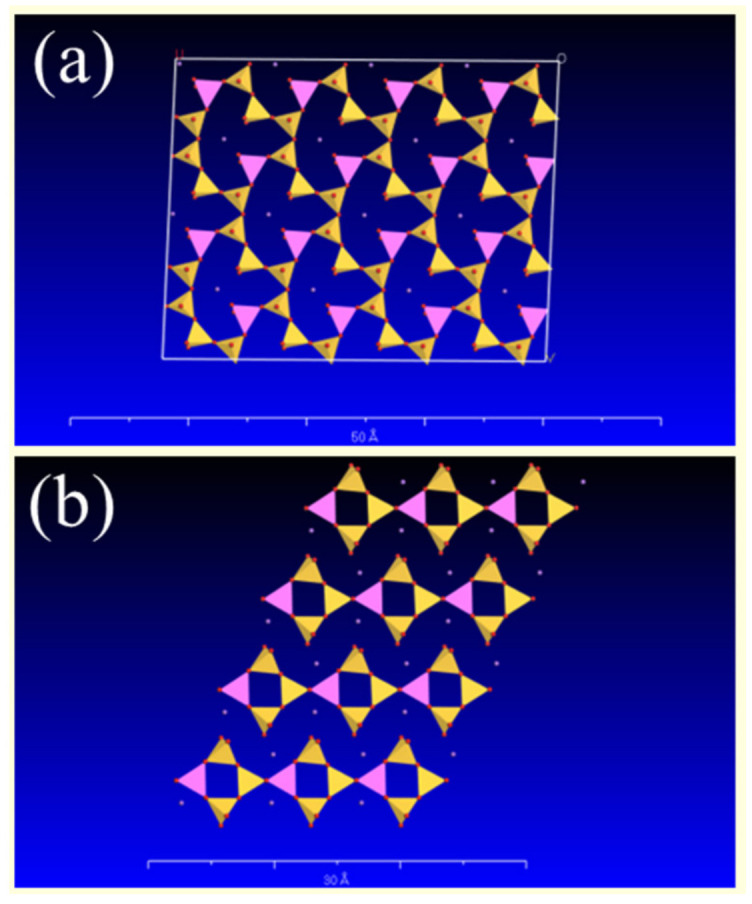
Top view of (**a**) the (002) and (**b**) (040) feldspar surfaces (pink is [AlO_4_], and yellow is [SiO_4_]).

To evaluate the extent of damage on the crystal faces caused by different organic acids, the I(002)/I(040) value is employed. A higher value indicates that the (040) face has been more severely affected compared to the (002) face (Figure 7), while a lower value suggests the opposite. Following treatment with an organic acid, the I(002)/I(040) value increased (Table 4), indicating that the (040) face of the crystal in alkali feldspar (albite and microcline) is more susceptible to attack due to a higher cavity content (Figure 7).

The order of organic acid solubilization ability on the (040) face is as follows: complex acid > citric acid > oxalic acid. Citric acid exhibits a greater ability to dissolve Si, making it more effective in dissolving the (040) face of the feldspar when compared to oxalic acid. The composite solution demonstrated the highest dissolution rate for the (040) face of the feldspar. Citric acid has a greater tendency to dissolve Si, while oxalic acid tends to dissolve Al more than citric acid, as illustrated in Figure 3 and Figure 4. The complete dissolution of the four-noded ring was largely facilitated by the simultaneous dissolution of adjacent [AlO_4_] and [SiO_4_] sites. When oxalic and citric acids are both present, they concurrently dissolve the Si and Al sites in the four-noded ring, accelerating the overall crystalline dissolution process.

**Figure 8:**
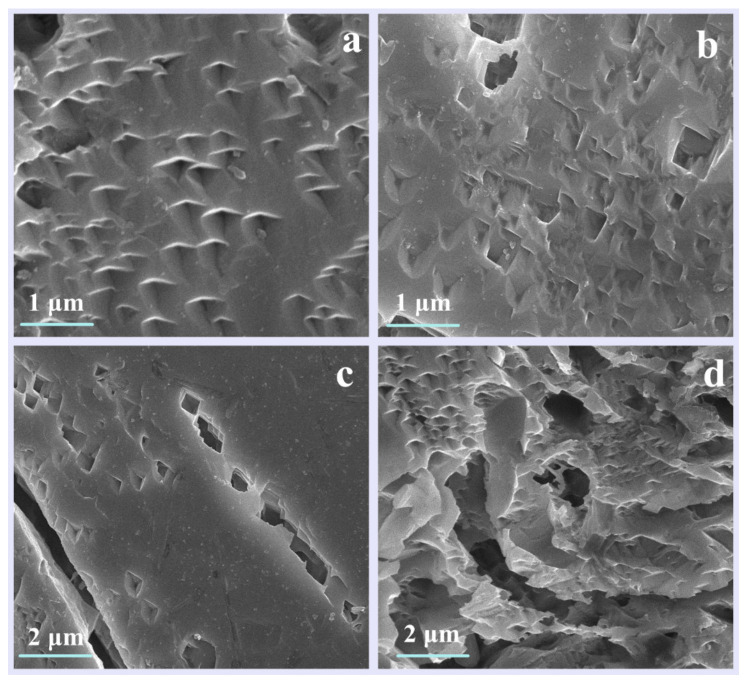
The SEM images of alkali feldspar after treatment with composite organic acids for (**a**) 72 h (**b**) 120 h (**c**) 240 h (**d**) 720 h.

The morphology of feldspar treated with composite organic acids for different durations are shown in Figure 8. After 72 h of treatment, the mineral surface exhibited almond-shaped corrosion pits with a preferred orientation. As the treatment time increased to 120 h, the area and depth of these pits increased, and rhombic corrosion pits appeared on the surface. After 240 h, adjacent rhombic corrosion pits connected to form channels. At the 720-h mark, the corrosion channels had enlarged, and corrosion grooves with corrugated and jagged edges had formed. Additionally, small, independent pits such as almond-shaped and diamond-shaped pits appeared on the edges and grooves, which were the result of the LMWOAs attacking new reactive sites. The specific surface area of the feldspar mineral increased in the following order: S (720 h) > S (240 h) > S (120 h) > S (72 h), as shown in Table 5. This indicates that a greater number of pits on the mineral’s surface leads to a larger specific surface area.

**Figure 9:**
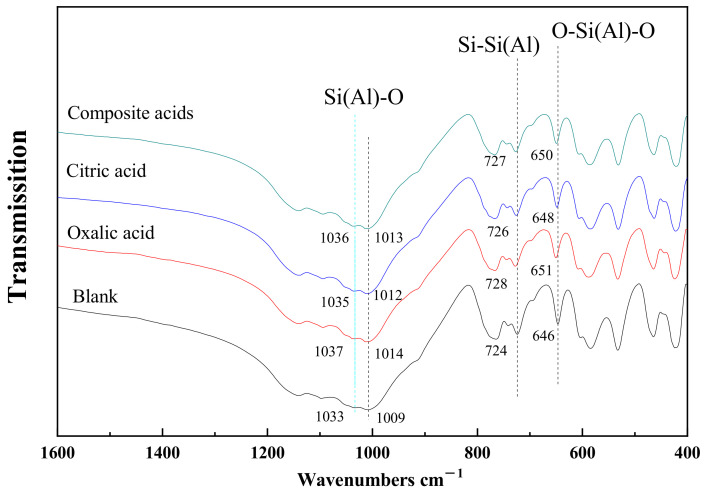
The FTIR spectra of feldspar after treatment with different organic acids.

Figure 9 displays the Fourier-transform infrared (FTIR) spectra of feldspar treated with various organic acids. Overall, the characteristic bands of alkali feldspar showed negligible changes after treatment with organic acids. This can be attributed to the strong Si-O and Al-O bonds in feldspar, which are highly resistant to extensive disruption by low concentrations of organic acids. Consequently, the chemical bonding environment of the crystal remained largely unchanged.

Fourier-transform infrared spectroscopy, an extremely sensitive tool for testing the chemical bonding of substances, revealed similar changes in the characteristic bands of alkali feldspar after organic acid treatment. The organic acid treatment of the feldspar mineral resulted in a shift towards higher wave numbers in the Si (Al)-O (1033 cm^−1^ and 1009 cm^−1^) and Si-Si(Al) (724 cm^−1^) stretching vibrational bands, and the bending vibrational band O-Si(Al)-O (646 cm^−1^) [27,28]. This implies an increase in the vibrational frequency of the Si-O bond associated with Al. This increase is attributed to the non-stoichiometric dissolution of Si and Al (Si/Al < 3) in the tetrahedral unit composed of one [AlO_4_] tetrahedron and three [SiO_4_] tetrahedra (Figure 7). Consequently, there is a higher presence of strong Si-O bonds in the tetrahedral ring compared to Al-O bonds, leading to an increased electron cloud density of the Si-O bonds. When comparing the IR vibrational profiles of feldspar after oxalic and citric acid treatment, it can be observed that the band shifts associated with Si(Al)-O and Si-Si(Al) vibrations were smaller after citric acid treatment, indicating a reduced non-metric dissolution of Al in the crystal structure. This suggests that citric acid is more reluctant to attack the Al-O bond than oxalic acid. Additionally, citric acid exhibits a stronger ability to break Si-O bonds compared to oxalic acid.

**Figure 10:**
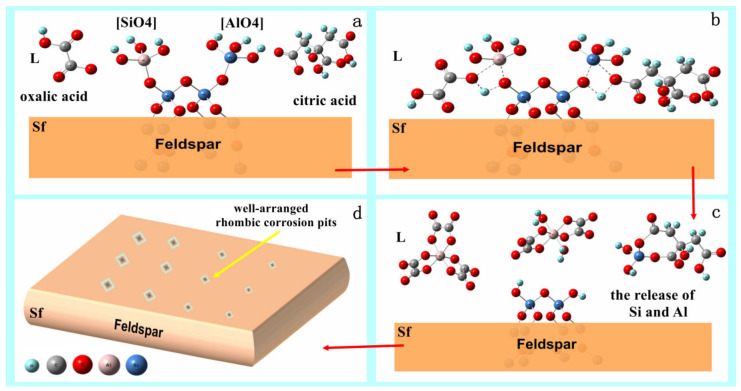
The schematic diagram of the dissolution mechanism of alkali–feldspar in the presence of composite organic acids. ((**a**), the organic acid ionizes in the aqueous solution, producing hydrogen ions and carboxylate organic acid anions (R-COO-); (**b**), The ions diffuse and adsorb to the Si and Al sites on the feldspar surface, forming surface complexes; (**c**), the breakage of the Si(Al)-O bond, and the release of Si and Al from the minerals; (**d**), the formation of well-arranged rhombic corrosion pits on the feldspar surface.)

The dissolution mechanism of feldspar by oxalic acid and citric acid can be derived from the above analysis as follows:

(1) At the beginning of the reaction, the organic acid ionizes in the aqueous solution, producing hydrogen ions and carboxylate organic acid anions (R-COO-) (Figure 10a). These ions then diffuse and adsorb to the Si and Al sites on the feldspar surface (Figure 10b), forming surface complexes denoted as Sf-Si (Al) -Lx (Sf represents the mineral solid phase, L represents the organic acid molecule, and x represents the number of organic acid molecules adsorbed to the same Si (Al) site).
(2) At the solid-liquid interface, the organic acid molecules polarize the Si(Al) in the adsorption site, causing a rearrangement of the Si(Al)-O bond electron cloud, leading to elongation and breakage of the Si(Al)-O bond (Figure 10c).
(3) The dissolution process is influenced by the variable density of Si and Al arrangement in the individual crystal faces of feldspar, as well as by the selective orientation of citric acid and oxalic acid to dissolve Si and Al. As a result, the (040) faces of microline and albite in feldspar are preferentially dissolved under the influence of the organic acids, followed by (002).
(4) The synergistic effect of the composite acids leads to the rapid formation of well-arranged rhombic corrosion pits on the feldspar surface (Figure 10d). These corrosion pits increase in number with reaction time, resulting in an increase in the specific surface area of the feldspar.

## 4. Conclusions

The dissolution behavior of alkali feldspar in different low-molecular-weight organic acids (LMWOAs) was investigated by analyzing the concentration of Si and Al and the changes in feldspar. It was found that citric acid was extracted Si from feldspar more easily than oxalic acid, while oxalic acid was more effective in extracting Al from feldspar than citric acid. The composite organic acid exhibited the strongest dissolving capacity for Si and Al, with values of 538 μM and 287 μM, respectively. The release of an Al atom from alkali feldspar was not followed by the release of three Si atoms. The data of Si and Al concentration fitted well with a first-order equation in the organic acid solution (R^2^ ≥ 0.992). LMWOAs were found to effectively alter the crystal structure of albite and microcline in feldspar. The (040) face of feldspar was more susceptible to attack than the (002) face in the presence of organic acids. The non-metric dissolution of Si and Al (Si/Al < 3) resulted in a shift towards higher wave numbers in the Si (Al)-O, Si-Si(Al), and O-Si(Al)-O bonds. In the presence of organic acids, anions diffused, adsorbed, and polarized the Si(Al) sites on the feldspar surface, thereby extracting Si or Al. The treatment with organic acids led to changes in the microstructure of the feldspar surface over time. Almond-shaped nano corrosion pits, rhombic corrosion pits, and channels were sequentially formed. The specific surface area of feldspar increased with time. This study provides insights into the dissolution mechanisms of feldspar in different LMWOAs and the production of activated feldspar powders in an environmentally friendly manner.

## Figures and Tables

**Table 1:** Chemical composition (wt.%) of the minerals.

Sample	SiO_2_	Al_2_O_3_	K_2_O	Na_2_O	Fe_2_O_3_	CaO	TiO	Fe_2_O_3_	LOI ^1^
the minerals	65.75	17.32	6.40	4.38	0.32	0.11	0.06	0.32	5.34

LOI ^1^ = Loss on ignition.

**Table 2:** The kinetic parameters of Si released from the sample in the different organic acids.

The Solution	$C_{t}=a+bt^{1/2}$		$C_{t}=a+blnt$		$C_{t}=a(1-exp(-kt))$	
	b	R^2^	b	R^2^	b	R^2^
Composite acid	31.67	0.986	202.41	0.893	1.96 × 10^−3^	0.992
Citric acid	13.83	0.985	90.61	0.925	1.21 × 10^−3^	0.992
Oxalic acid	8.83	0.988	57.07	0.898	1.08 × 10^−3^	0.997
Blank	0.61	0.959	4.05	0.963	8.60 × 10^−4^	0.972

**Table 3:** The kinetic parameters of Al released from the sample in different organic acids.

The Solution	$C_{t}=a+bt^{1/2}$		$C_{t}=a+blnt$		$C_{t}=a(1-exp(-kt))$	
	b	R^2^	b	R^2^	b	R^2^
Composite acid	16.28	0.985	104.80	0.889	9.55 × 10^−4^	0.997
Citric acid	5.91	0.969	37.66	0.855	4.58 × 10^−4^	0.993
Oxalic acid	5.20	0.977	33.29	0.869	5.84 × 10^−4^	0.996
Blank	0.24	0.992	1.59	0.946	2.21 × 10^−4^	0.993

**Table 4:** The value of I(002)/I(040) of microcline and albite in the feldspar mineral before and after treatment.

Cystal	Raw Materials	Oxalic Acid	Citric Acid	Composite Organic Acids
Microcline	1.65	2.83	3.37	3.66
Albite	1.51	2.08	3.03	3.39

**Table 5:** The special surface area of feldspar after treatment with composite organic acids for different times (h).

Treatment Time (h)	0	72	120	240	720
SSA (m^2^/g)	1.91	2.38	2.57	2.83	3.25

## Data Availability

The data presented in this study are available on request.
